# Against the Odds: A Structural Equation Analysis of Family Resilience Processes during Paternal Incarceration

**DOI:** 10.3390/ijerph182111592

**Published:** 2021-11-04

**Authors:** Amy A. Morgan, Joyce A. Arditti, Susan Dennison, Signe Frederiksen

**Affiliations:** 1 Department of Family Science, School of Public Health, University of Maryland, College Park, MD 20742, USA; 2 Department of Human Development and Family Science, Virginia Tech, Blacksburg, VA 24061, USA; jarditti@vt.edu; 3 School of Criminology and Criminal Justice, Griffith University, Brisbane, QLD 4111, Australia; susan.dennison@griffith.edu.au; 4 Children and Education Department, VIVE Danish Center for Social Science Research, 1052 Copenhagen, Denmark; signefred78@gmail.com

**Keywords:** parental incarceration, paternal incarceration, family processes, family resilience, structural equation modeling, mass incarceration, Danish families

## Abstract

On any given day, approximately 2.1 million children in Europe have an incarcerated parent. Although research indicates that material hardship is associated with parental incarceration, and particularly paternal incarceration, little is known about family processes that may mitigate the harmful effects of such hardship on children with an incarcerated parent. Guided by a resilience framework, this study examined how family processes mediate the effects of material hardship on youth academic adjustment within the context of paternal incarceration. Using Danish data that assessed key family constructs, structural equation modeling was used to perform a mediational within-group analysis of primary caregivers (n = 727) to children with an incarcerated father. Results indicate that although social support and parenting skills did not yield mediating effects, caregiver mental health strongly mediated the effects of material hardship on youth academic adjustment during paternal incarceration. Findings suggest that economic conditions, as well as caregiver mental health symptoms, are important areas of intervention that may promote family-level resilience for youth of an imprisoned father. We conclude with research and practice recommendations to advance our understanding of resilience among families with an incarcerated parent.

## 1. Introduction

Although the rising global incarceration rate has remained fairly commensurate with rising population rates (i.e., 24%), changes in prison population rates since 2000 vary considerably across countries [1]. The United States (U.S.) still maintains the highest prison population rate (i.e., 655 per 100,000), but as incarceration rates begin to slow in the Americas, Europe’s prison population rate has slightly increased (i.e., by 3%; 192 per 100,000) since 2000 [1]. Across Europe, the number of estimated children (i.e., 2.1 million) with an incarcerated parent is equivalent to that of a small country [2]. Rising and maintained rates of prison populations, and the sheer number of children experiencing parental incarceration (PI), warrant consideration of the many families impacted globally by carceral practices. Nevertheless, empirically examining the effects of PI on the family has been historically challenging due to a general lack of family level PI data, as well as methodological barriers to conducting research with incarcerated persons, and by extension their families [3,4,5]. Although much remains to be known about the effects of PI, existing research clearly shows that PI broadly generates adverse outcomes for youth and families [2,3]. Furthermore, the negative effects of having an incarcerated parent, and in particular paternal incarceration, are fairly consistent across many Westernized nations including the U.S., United Kingdom, Norway, the Netherlands, and Denmark, despite differing sociopolitical contexts (e.g., social welfare, access to healthcare, “tough on crime” policies, etc.) and criminal justice practices (e.g., sentence length, prison conditions, rehabilitation approaches, day or weekend release practices, etc.) [6]. As new theories and data emerge, opportunities to test them must be seized to advance our understanding of how PI shapes youth and families. Given the abundance of risks and adverse outcomes experienced by families with an incarcerated parent, it is imperative that we focus on understanding protective factors and processes that mediate against negative outcomes and that possibly promote resilience. 

In this paper, we present compelling findings that highlight possible mechanisms of family resilience in the context of paternal incarceration. Our findings provide meaningful contributions to the PI literature in several distinct ways. First, our data contained a focus on family process perspectives. Family-level data examining the effects of PI remain relatively rare, rendering our research a unique opportunity to consider paternal incarceration in a family context. Second, our data were collected in Denmark. PI research among Danish families is still developing, and existing literature indicates that PI data from Denmark offer comparative insights to other Westernized countries [6]. Finally, this study was among the first to concurrently examine both risk and resilience in a robust manner using family-level data, offering promising theoretical and intervention-based possibilities for families with an incarcerated parent. To best situate our findings, we first provide an overview of the Danish context. 

## 2. Danish Context

At present, approximately 5144 Danish children (i.e., about 6%) have an imprisoned father [2,7]. Youth and family experiences of paternal incarceration differ greatly based on existing risk and protective factors (e.g., socioeconomic status, family stability, social safety net, etc.), as well as criminal justice trends and practices (e.g., frequency, duration, and location of paternal incarceration) [6,7,8,9]. In Denmark, rates of serious criminal activity and convictions are relatively low compared with other Westernized nations, such as the U.S., which serves as a comparison of extreme incarceration trends [7]. For example, the homicide rate in the U.S. (3.8 per 100,000) is 54 times higher than that of Denmark (0.7 per 100,000) [7]. However, rates of “less serious crimes” (e.g., traffic violations) do not vary greatly between these two countries [7]. 

The justice systems between Denmark and the U.S. are distinct in six ways, as summarized by Wildeman and Andersen (2017), and contribute to our understanding of how these countries differ [9]. First, Denmark generally has harsher sentences than the U.S. for serious traffic violations (e.g., driving under the influence) [9]. Second, carceral sentences are generally much shorter in Denmark across all crimes [9]. Third, individuals incarcerated in Denmark are eligible for parole after two-thirds of their sentence has been served, whereas Americans tend to serve higher proportions of their sentences [9]. Fourth, Denmark uses non-custodial alternatives to traditional incarceration, such as community service and electronic monitoring, at much higher rates than other countries [9]. Fifth, and perhaps most notably, about one-third of Danish prisons are “open” [8], in which incarcerated persons can leave for skill promoting opportunities such as employment and work. Finally, the practice of incarcerating people in local jails (i.e., closer to home) for shorter sentences does not occur in Denmark. Indeed, Danish incarcerated persons with short sentences may serve time in carceral settings that are very far from home [9]. Even when contact and visiting experiences are possible, experiences vary greatly in quality [8].

Crime rates are only part of the whole incarceration picture. In addition to lower rates of imprisonment and generally humane prison conditions, Denmark also has an extensive welfare system fostering robust safety nets that might compensate for the effects of PI on children and families [6,7,8,9]. Nonetheless, emerging research suggests that despite an expansive welfare system, PI may still adversely impact Danish children’s education and other developmental outcomes. In this way, if PI is a mechanism of risk for families with significant social welfare resources, it is likely we would find the same outcomes, if not worse, in countries with less socialized resources such as the U.S. Regardless of differences across countries, PI research outcomes (i.e., foster care placement and youth well-being) have been remarkably consistent across transnational criminal justice contexts [8], including the U.S. and Denmark. Danish studies may also offer useful insights for jail/pre-trial sentences in other countries, as the average duration of prison sentences in Denmark are shorter in nature [9]. Given that the present study is not examining predictors or outcomes of incarceration, but rather family resilience processes during incarceration, we argue that findings from this research may inform our general understanding of family resilience processes when a parent is incarcerated. Indeed, PI scholars assert that Danish studies examining processes present during PI can provide important comparative insights that are relevant and applicable to other jurisdictions and may point to even greater effects in countries with less social welfare resources [6,7,8,9]. 

## 3. Risk and Resilience during Parental Incarceration

The era of mass incarceration has generated a destructive wake of consequences for youth and families [10,11,12,13,14,15,16]. Children of incarcerated parents often face unique risk factors that their age-matched counterparts do not. For example, many children with an incarcerated parent will enter the criminal justice system, suggesting a systemic and intergenerational pattern of incarceration [16]. Furthermore, children with an incarcerated parent also tend to have higher rates of mental illness, trauma, antisocial behavior, academic challenges, poor physical health, profound poverty, parental substance use, stigma-laden interactions, and homelessness [10,17,18,19,20,21,22]. Research highlighting poor outcomes among families experiencing PI are consistent across developed countries, particularly the United States and Europe [7,16,23,24,25]. PI often equates with significant alterations in family roles and responsibilities, particularly if the incarcerated parent provided meaningful contributions to the family prior to confinement [14]. Paternal incarceration has been linked to an increase in single parent households [26] and can have profound implications for those remaining caregivers [17]. The non-incarcerated caregiver (e.g., biological parent, extended family, foster parent, etc.) may experience psychological and financial distress, role strain as a caregiver, and overall family instability [15,19,27]. Consequently, negative child outcomes may be a function of family-level processes during PI, and especially maternal caregiving during paternal incarceration [27,28]. For example, research indicates that children with incarcerated parents are more likely to experience socioemotional problems, including internalizing disorders such as depression and anxiety [16,20] and externalizing disorders such as such as delinquent conduct [29]. However, findings vary by child gender and the type of crime associated with the parent’s sentencing [30]. Furthermore, families with an incarcerated parent are often caught in an impossible cycle where cumulative disadvantage predicts carceral involvement [8,30], and carceral involvement worsens economic and family well-being over time [14,18,19,20,21,22]. 

Historically, much of the PI literature has highlighted risks and determinants of pathology (e.g., mental health and substance use problems, risks for offending, etc.) for youth with an incarcerated parent [19,22]. Based on the extant literature, we can reasonably conclude that PI overwhelmingly produces increased risks for youth and families across the world [12,13,14,15,16,17,18,19,20,21,22,23,24,25,26,27,28,29,30,31,32,33], even among countries with extensive social safety nets [6,7,8,9,25,28]. Nevertheless, there is a significant lack of research that uses a resilience framework to examine family resilience processes in the context of PI [31,33]. Here, we define family resilience as the process by which families withstand and rebound from serious life challenges [34,35,36]. To assess the degree of family system resilience, Patterson (2002) stated that the empirical method must involve at least two family members, with a specific focus on family relationships (i.e., linkages and processes between family members) [37,38]. Masten and Coatsworth (1998) provided further family resilience research criteria: (1) the presence of a contextual risk (e.g., material hardship) that we would expect to cause family crisis and (2) a need to understand protective processes that prevent otherwise expected poor outcomes [39]. The lack of PI research on family resilience processes may be, in part, due to a lack of within-group resilience research, which may be better suited to identify specific resilience processes and attributes than comparative between-groups research (i.e., control or comparative group) [31,33].

Family resilience process research broadly provides an opportunity to examine the systemic interdependence of family members in shaping one another’s experiences and is crucial to better understanding the effects of PI on the family as a whole [15]. Advancing this line of research is imperative to our understanding of how youth and families may fare well, or even excel, in the context of PI [31,33,35]. Indeed, PI scholars have identified an urgent need for understanding resilience processes among families with an incarcerated parent [31,33]. Resilience research findings have the potential to inform intervention programs just as much, if not more, than pathology-based research of youth experiencing PI [31,33,35]. However, resilience research within contexts of PI researchers may be limited by a general lack of data containing key family-level constructs central to resilience frameworks, such as parenting quality and practices associated with the nonincarcerated caregiver. As new family-level PI data emerge, we must leverage opportunities to better understand family resilience mechanisms among families with an incarcerated parent. Toward that end, this paper presents findings from a within-group analysis examining mediating resilience processes of Danish families impacted by paternal incarceration. We use a risk and resilience framework (i.e., the Family Inequality Framework; [35]) to advance our understanding of how these families fare well despite significant adversity. 

## 4. Family Inequality Framework

**Figure 1:**
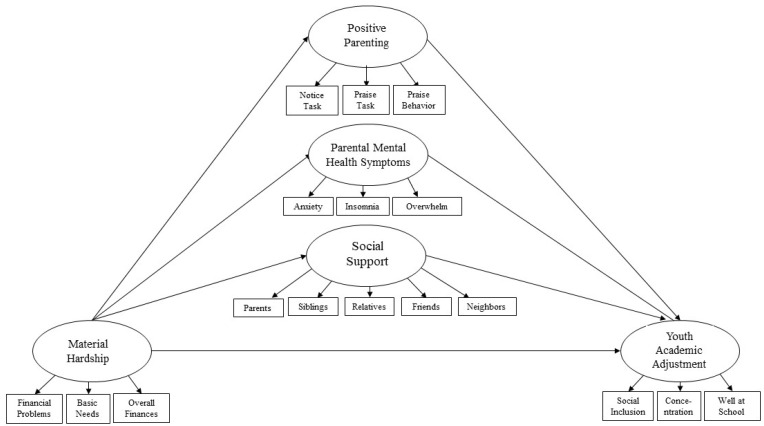
Conceptual Model.

Informed by Family Stress and Resilience Theory [36,37,38], the Family Inequality Framework (FIF) [35] conceptualizes PI as an ongoing stressor that influences critical family processes such as parenting and youth academic adjustment. Within the FIF, material hardship (i.e., economic hardship) is a key mechanism of stress [35]. Family coping (e.g., social support, positive parenting, and stable caregiver mental health) acts as resilience processes that buffer the impact of these stressors in families experiencing PI [35]. Although the risks inherent in PI have been documented extensively, less is known about the degree to which family processes represented in the FIF broadly promote family resilience during PI and specifically mediate the effect on youth academic adjustment [15]. Investigating mechanisms of risk and resilience in a family context may promote positive youth and family outcomes despite material hardship during PI [31,33]. In this paper we present an empirical test of the theoretical FIF by examining how family processes, such as caregiver mental health, positive parenting, and social support, mediate the effects of material hardship on youth outcomes during paternal incarceration. These processes are visually represented in the conceptual model (Figure 1).

### 4.1. Material Hardship

Incarcerated persons are more likely to experience poverty, reside in low socioeconomic status neighborhoods, and hold low-wage positions before and after incarceration [40,41]. Children of incarcerated parents are significantly less likely to have financial support (e.g., child support and general financial assistance) than children who have not experienced PI [19]. Incarceration can further exacerbate material hardship through loss of income, legal fees, and/or barriers to re-entering the labor market with a criminal record [42], perpetuating existing economic inequities and resulting in cumulative disadvantage. The relationship between PI and cumulative disadvantage may be especially true in countries with less robust social safety nets (e.g., social welfare and healthcare) [43]. These findings suggest that incarcerated individuals likely experience significant material hardship, which may have profound direct and indirect implications for their families and children. Conger and colleagues (2010) proposed that economic (i.e., material) hardship acts as a conduit for risk factors in family relationships; material hardship not only affects child development directly but also transmits effects of material hardship through parental experiences (i.e., parenting, coping, stress etc.) [44]. The FIF indicates possible protective processes (e.g., social support, parenting, and caregiver mental health) that may mediate the effects of material hardship and contribute to resilience processes [35].

### 4.2. Family Resilience Processes

#### 4.2.1. Social Support

Social support can act as a protective factor during times of stress and can mediate the harmful effects of economic and psychological distress during PI [43,45,46,47,48]. Social support may be experienced as financial, emotional, or instrumental help by friends, family, and community members [48]. For many families, incarceration means an abrupt change in financial, household, and parenting responsibilities [46,47,49]. Other types of family loss (e.g., deployment, death, etc.,) are usually accompanied by support and sympathy for the involuntary nature of the structural family change [14]. Incarceration, however, does not tend to draw the same type of social support; indeed, the social stigma surrounding incarceration may impede a family’s ability to [openly, publicly] grieve the ambiguous loss often inherent in PI. As Arditti (2012) states, “There are no casseroles brought to the house for the ‘prison widow’ and her children. There is no government assistance or formal recognition that a significant loss has occurred in the family that will bear heavily on children’s welfare” (p. 112) [15]. Mothers are most burdened by paternal incarceration, yet may have limited access to, or offers of, support [50]. From a family stress perspective, resources are needed in times of strain to promote family adaptation. One study with incarcerated parents demonstrated that social support had a mediating effect on the parent’s psychosocial functioning and depressive symptoms [51]. However, Turney and Wildeman’s (2013) findings demonstrate a “double strike” within the context of paternal incarceration in that the availability of instrumental support may decrease while the need for support increases [47]. However, further research is needed to examine the role social support may play in mediating the effects of material hardship on youth outcomes during PI.

#### 4.2.2. Caregiver Mental Health

Poor caregiver mental health is consistently linked with negative child and family outcomes across a variety of domains [41] and may be a spillover effect from socioeconomic struggles related to paternal incarceration [52]. Conversely, research suggests that the mental well-being of the non-incarcerated parent/caregiver may mitigate adverse outcomes during PI [27,53]. The hypothesized relationship between the mental well-being of PI caregivers and consequences for youth is evident in studies that demonstrate positive prosocial and well-being outcomes for children of parents with little or well-managed stress [54]. For example, Dallaire and Aaron (2010) reported that children’s responses to PI were influenced by the mental well-being of their caregivers [55]. Although this area of research requires further advancement, scholars agree that the importance of the caregiving context (i.e., caregiver mental health) is imperative to the well-being of children with an incarcerated parent [53,54]. Consequently, caregiver mental health may serve as a family resilience process in which child well-being is a function of parental wellness despite other contextual stressors such as material hardship [26,49]. Although previous research has indicated the general importance of having a stable caregiver during PI [52,53,56,57], further research is needed to examine caregiver mental health as a specific mediating resilience process with families experiencing PI.

#### 4.2.3. Positive Parenting

High quality parenting has begun to be conceptualized as a resilience process that mediates the potential effects of PI [14,35,53,58,59]. However, due to a dearth of empirical research about positive parenting behaviors of the nonincarcerated caregiver in contexts of PI, we must extrapolate the importance of positive parenting by considering its opposite—that is, studies that examine parenting stress and less optimal parenting strategies. For example, research suggests an association between paternal imprisonment and high maternal parenting stress [52,59]. A recent examination of heterogeneity in maternal stress during paternal incarceration found that experiencing material hardship and poorer well-being prior to incarceration was associated with no apparent change in parenting stress, yet if parenting stress was high before incarceration it remained high during incarceration [45]. Further, research by Wildeman and colleagues (2012) suggested that changes in material hardship were associated with increased parenting stress and worsened mental health, suggesting overall negative spillover effects across financial and socioemotional domains [52]. Scholars have hypothesized that PI may impede effective parenting skills for the non-incarcerated parent because of economic strain and psychological distress [20]. For example, research suggests that paternal incarceration is associated with maternal authoritarian parenting practices and neglect [20]. Additionally, these mothers tended to report increased experiences of material hardship as well as family instability [20]. Turney (2014) also found child neglect and maternal physical aggression to be positively associated with paternal incarceration [20], which may be explained, in part, by caregiver mental health and material hardship [20,45]. Within the FIF, material hardship during PI could exacerbate strain in the family relationships and on the caregiving parent, and result in collateral harm to the child through the primary caregiver’s parenting. Findings by Besemer and Dennison (2018) support this notion, indicating that parenting stress is likely a mediating factor in child adjustment during paternal incarceration [59]. Conversely, if parenting stress mediates hardship and child outcomes, the presence of positive (i.e., warm and authoritative) parenting practices could foster positive outcomes for youth with an incarcerated parent. Indeed, findings suggest that positive parenting experiences may be among the most important parental processes for families with an incarcerated parent, even in the context of material hardship [52]. Nevertheless, further research is needed to examine the nuances of parenting effects within families experiencing PI. For example, the presence of positive parenting (i.e., a family strength) is not the same as the absence of pathology [31] and thus warrants further examination. 

### 4.3. Youth Academic Adjustment

Although primarily deficit-focused, an emerging literature is beginning to document evidence of children’s competence in contexts of PI [33]. These competencies are developmentally specific and linked to age salient tasks such as attachment and school success [57]. Therefore, for school-aged youth, academic adjustment is a critical area of concern [60]. PI has been associated with poor functioning across multiple academic domains, including class ranking, truancy, class failure/pass rates, education incompletion, and failure to complete standardized exams [61]. College graduation rates were also found to be significantly lower for children of incarcerated parents than children with parents who were never incarcerated [61]. While academic performance appears to be consistently lower for children of incarcerated parents, findings on school dropout are mixed. Research by Cho (2011) indicated that adolescents are at a significantly higher risk for dropping out of school in the year that PI began [62]. Furthermore, consistent with Hagan and Foster’s (2012) findings, adolescents with an incarcerated parent are neither more nor less likely to drop out of school when attending school in an area of high rates of PI [60,61,62]. One potential explanation for this finding is that teachers may increase sympathy and lower expectations for children and adolescents experiencing PI more than other common reasons for poor academic performance [62]. Still, findings examining the association between PI and academic adjustment remain mixed [61], and scholars suggest that this may be a result of age, with academic failure compounding over time [60]. Examining youth academic adjustment in relation to PI makes sense, as it is generally understood to be a reliable indicator of well-being in later life [63] and may provide a multidimensional perspective on child development, as it examines skills across social, behavioral, and learning domains [61]. Consequently, we incorporated an under-researched developmental outcome that represents both child functioning and serves as a potential indicator of future adjustment.

## 5. Current Study

The present research examines the effects of PI on youth academic adjustment in an effort to model how family resilience processes may mitigate negative youth outcomes of paternal incarceration [31,33,35]. Furthermore, parental incarceration data containing family-level variables are uncommon. Existing data with a focus on family process perspectives are particularly attractive due to the historic methodological barriers to data collection with the incarcerated population [64]. The present study advances the PI literature in several ways. First, we explore youth academic adjustment in the context of paternal incarceration. Second, we shift the PI literature from a deficit-focus to a strengths-based focus by examining mediating resilience processes of families with an incarcerated father. At present, both youth academic adjustment and family resilience are underdeveloped research foci within the PI literature. Furthermore, our study presents findings from Danish families, which may address important questions about PI in Denmark and potentially provide comparative insights for other geographic contexts [65]. Finally, in examining the first two aims we provide an empirical test of the FIF [35], which theoretically situates resilience processes as a potential mediating effect of PI on youth outcomes. We employed a cross-sectional structural equation modeling (SEM) analysis to examine the degree to which family resilience processes mediated the effects of material hardship on youth school adjustment during paternal incarceration. Based on the extant literature and theory, we hypothesized the following: (H1; direct effect) material hardship will be negatively associated with youth academic adjustment, and (H2; indirect effect) the effects of material hardship on youth academic adjustment will be mediated (either partially or fully) by family resilience processes (i.e., positive parenting, parental mental health, and social support). It is our hope that findings from this study can inform the development of much needed family-level interventions aimed at mitigating risks and promoting resilience among families facing PI [54]. 

## 6. Method

### 6.1. Data Source

To test the proposed model (Figure 1), we conducted a secondary analysis of data from surveys randomly mailed to Danish families with an incarcerated father in 2015. Original data collection was conducted by the Danish Center for Social Science Research (VIVE) [66]. The survey used in this study contained caregiver-completed items about family stress, resilience processes, and youth academic adjustment outcomes. These items were instrumental in testing the influence of family resilience processes in youth adjustment. All respondents (n = 727) experienced incarceration of the child’s father within the three years prior to data collection. Length of sentence and type of carceral setting were unknown. 

### 6.2. Sample Characteristics

Although demographic information collected about survey participants was limited, basic sample parameters were as follows. Most respondent caregivers were female (n = 678; 93.3%), and nearly half (n = 366; 50.3%) of the children were female. In order of frequency, children were aged: 11 (n = 238; 32.7%); 13 (n = 204; 28.1%), 15 (n = 147; 20.2%), and 17 (n = 138; 19.0%). Most caregiver respondents identified as the child’s biological mother (n = 602; 82.8%). The remaining respondents identified as a foster-mom (n = 66; 9.1%), biological father (i.e., previously incarcerated within the prior three years) (n = 23; 3.2%), paternal grandmother (n = 6; 0.8%), maternal grandmother (n = 5; 0.7%), other caregiver type (n = 2; 0.3%), and step-mom (n = 1; 0.1%).

### 6.3. Measurement and Construct Development

Decisions about construct development and measurement were informed by the research team’s preliminary inspection of the Danish survey and existing descriptive data [65] in collaboration with VIVE. Description of the data can be found in Table 1. Internal reliability consistency analysis was performed to examine the indicators of each respective latent construct. Initial factor analyses were examined by each construct using a principal component analysis with varimax rotation. McDonald’s omega (ω), which is considered a more robust measure of reliability than Cronbach’s alpha (α) [66], was calculated to examine the reliability among construct indicators. Omega values indicated moderate to strong reliability (reported below within each corresponding construct), which was again confirmed in the confirmatory factor analysis (CFA). A CFA was conducted to examine how well each construct was measured by its respective manifest indicator. Mplus [67] was used to test the CFA. The measurement model demonstrated an overall good fit to the data based on the model fit indices. Specifically, the chi-square value ($\chi^{2}$ = 264.026, *p* = 0.0000, *df* = 109; [68]), RMSEA (0.04; [69,70]), CFI (0.94; [68]), TLI (0.92; [68]), and SRMR (0.04; [68]) were all within acceptable ranges, indicating that the hypothesized model was measured adequately. However, modification indices did indicate minor issues with the measurement of two constructs. Two of the original caregiver mental health items (i.e., chest pain and body aches) did not add to the overall measurement of the construct and were removed. Further, modification indices indicated that the construct social support would be better measured if items were not summed as one indicator. Consequently, the summed indicators were ungrouped and re-organized as five stand-alone items—specifically, support from respondents’ parents, siblings, general relatives, friends, and neighbors. Each of these indicators was represented by a single item inquiring about the frequency in which respondents were able to receive support from these individuals. The remainder of the hypothesized model stayed in its original form. After making adjustments informed by the CFA, all factor loadings were significant at the *p* < 0.001 level, indicating strong convergent validity [71]. Furthermore, all correlations were less than 1.0 among the factors, demonstrating evidence of discriminant validity [71]. The final SEM model, informed by measurement model modification indices, is represented in Figure 1. 

#### 6.3.1. Material Hardship

Material hardship was constructed by four survey items representing financial problems, basic needs, and overall finances. Items were then summed to better estimate the material hardship construct given the sample size. Example questions included, “Have you or your family struggled with the following due to financial problems?: Paying rent on time, paying bills on time, heating your home, etc.” Responses were either “yes” or “no”. Higher scores on material hardship equated with higher severity of financial burden, economic hardship, and difficulty meeting basic needs. Internal consistency reliability of material hardship items was excellent (ω = 0.93).

#### 6.3.2. Resilience Processes

Guided by the FIF, family resilience processes were conceptualized as mediators and were represented by three latent constructs in the conceptual analytic model (Figure 1). Factor analyses were conducted with each construct to assess validity and ensure that the constructs were measured appropriately. Positive parenting was measured by three 5-point Likert scale items asking participants the degree to which s/he “notices when their child does well”, “praises the child after she or he does a task well”, and “praises the child when s/he behaves well”. Higher scores equated with higher frequency of employing warm, authoritative parenting. Internal consistency reliability of the items within this construct was strong (ω = 0.82). Caregiver mental health was measured by five 3-point Likert scale items inquiring about the extent of participants’ mental health-related symptoms. Items in this construct asked participants if they experienced anxiety, insomnia, and a general feeling of being overwhelmed in the last year. Lower values indicated an absence or low prevalence of symptomology, while a higher value indicated a presence of troubling symptomology. Lower totaled scores equated with a positive caregiver mental health. Internal consistency reliability within this construct was moderate (ω = 0.66). To measure social support, participants were prompted with “If you need help with practical things, you can expect support from”, followed by response options: parents, siblings, general relatives, friends, and neighbors. Participants responded based on a 5-point Likert scale (i.e., “never” to “always”). A higher overall value represented a higher degree of support within the respective social support indicator. Internal consistency reliability of the items within the social support construct was moderate (ω = 0.68).

#### 6.3.3. Youth Academic Adjustment

The endogenous variable was represented by three items: social inclusion, ability to concentrate, and doing well at school overall. Each indicator was measured by 5-point Likert scale items assessing the caregiver’s perception of the youth’s academic experiences. Higher scores equated with a stronger degree of youth academic adjustment. Internal consistency reliability of the items within this construct was strong (ω = 0.71).

### 6.4. Data Analysis

SEM via MPlus [67] was used to test the theoretical model (Figure 1) using maximum likelihood (ML) estimation. SEM accounted for measurement error, thereby increasing power and reducing the potential for biased effects [72,73]. Consistent with the SEM mediation analysis procedures described by Preacher et al. (2007), we used bootstrapping to test specific mediation effects (i.e., caregiver mental health, parenting quality, and social support) within the model [74]. Using Monte Carlo simulation, a priori and post hoc power analyses were conducted to ensure sufficient statistical power. Fit indices were used to determine how well the model fit the data. 

## 7. Results

A total of four cases were deleted listwise, as all four participants opted out at the beginning of the survey, leaving the remainder of items unanswered. After the exclusion of these cases, the total sample size was *n* = 723. All variables had less than 10% of missing data, justifying the use of the maximum likelihood estimation method [75]. Bivariate correlations of variables and indicators were calculated prior to model specification (Table 2). The majority of the correlation coefficients were statistically significant at the *p* < 0.05 level. A confirmatory factor analysis established that latent constructs were measured well by their corresponding indicators. Univariate and multivariate data normality were confirmed for the remaining variables and the overall model, as indicated by skewness and kurtosis values falling within normal ranges (Table 1). Results were interpreted using parameter estimates for the final model (Table 3) and structural model standardized coefficients (Table 4). The causal SEM model demonstrated an overall good, consistent fit to the data based on the model fit indices. The chi-square value ($\chi^{2}\;$= 266.652, *p* = 0.000, *df* = 109; [68]), RMSEA (0.04; [69,70]), CFI (0.94; [68]), TLI (0.92; [68]), and SRMR (0.04; [68]) were all within acceptable ranges. 

### 7.1. Power Analyses

Monto Carlo simulations were employed in Mplus [67] statistical software to examine statistical power. Power is considered acceptable at the value of 0.80 and above [70]. A priori power results indicated that the statistical power for the study sample size (n = 723) would be 1.00 for detecting direct effects and associated indirect effects for both large main effect sizes (0.50) and medium main effect sizes (0.30). However, for small main effect sizes (0.1), there would be insufficient power to detect direct effects (0.65–0.60) and associated indirect effects (0.19–0.16). Post hoc power analyses indicated strong statistical power for all non-zero direct effects (1.00–0.94), except for the direct effect between positive parenting and academic adjustment (0.69). Furthermore, results for the full structural model indicated strong statistical power for both the sum of indirect effects (0.99) and indirect effects of caregiver mental health (0.99); statistical power for the indirect effects of positive parenting (0.01) and social support (0.07) were very low, which can be explained by the indirect effect sizes being zero.

### 7.2. Structural Model Results

#### 7.2.1. Direct Effects

**Figure 2:**
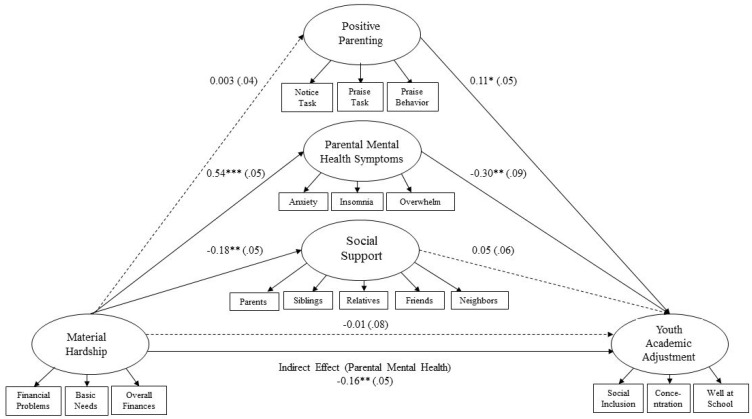
Structural Equation Analytical Model Results. * Correlation is significant at the 0.05 level (2-tailed). ** Correlation is significant at the 0.01 level (2-tailed). *** Correlation is significant at the 0.001 level (2-tailed).

The final model indicated several significant pathways among latent constructs, presented in Figure 2, as well as in Table 2 and Table 3. Standardized coefficients were used to scale construct values using a metric that is more easily interpreted. Material hardship yielded direct positive effects on caregiver mental health (β = 0.54, *p* < 0.001), with higher levels of material hardship resulting in increased levels of mental health symptomology issues for caregivers. Furthermore, material hardship had direct negative effects on social support (β = −0.178, *p* < 0.001), indicating that a more severe degree of material hardship predicted lower overall social support from respondents’ parents, siblings, other relatives, friends, and neighbors. Conversely, material hardship did not have a significant effect on either youth academic adjustment (β = −0.014, *p* = 0.861) or respondents’ positive parenting (β = 0.003, *p* = 0.952). Caregiver mental health symptomology had a significant direct negative effect (β= −0.301, *p* < 0.001) on youth academic adjustment, indicating that higher levels of poor caregiver mental health symptomology were associated with lower levels of academic adjustment. Furthermore, positive parenting had a significant direct positive effect (β = 0.115, *p* < 0.05) on youth academic adjustment, suggesting that parenting practices of noticing, encouraging, and praising good behavior predicted better overall academic adjustment. Respondent social support did not have a significant direct effect (β = 0.051, *p* = 0.394) on youth academic adjustment. Caregiver mental health was the only variable to have a significant direct effect between both the exogenous (i.e., material hardship) and the endogenous (i.e., youth academic adjustment) variables.

#### 7.2.2. Indirect Effects

Of the three mediating variables (i.e., positive parenting, caregiver mental health, and social support), there was considerable variation in the degree to which these variables interacted with the direct effect of material hardship on youth academic adjustment, lending partial confirmation to our second hypothesis (H2). Although positive parenting (β = 0.000, *p* = 0.956) and social support (β = −0.009, *p* = 0.419) yielded no mediating effects, caregiver mental health strongly mediated the relationship between material hardship on youth academic adjustment. Indeed, the most noteworthy finding was the significant indirect negative effect (β = −0.164, *p* < 0.01) that caregiver mental health symptomology had on the relationship between material hardship and youth academic adjustment. Despite an insignificant direct effect of material hardship on youth academic adjustment, results demonstrate that this relationship became significant to the degree that respondents endorsed mental health symptoms such as anxiety, insomnia, and a general feeling of being overwhelmed.

## 8. Discussion

Given evidence of the adverse impact of PI on children worldwide, it is critical that we examine both risk and resilience processes of families impacted by incarceration. While much of the existing PI research focuses on risks, the present study examined the mediating role of family resilience processes, advancing the PI literature from deficit-based between-groups comparisons to resiliency-based within-groups analyses [33]. Our findings suggest that, for Danish families with an incarcerated father, stronger caregiver mental health mediates the otherwise adverse effects of material hardship on youth academic adjustment. Our findings are especially notable given Denmark’s expansive social welfare system and its potential to mitigate adverse outcomes for families with an incarcerated parent [6,7,8]. It is possible that the impact of caregiver mental health as a mechanism in promoting family-level resilience during PI is even more pronounced in countries with less robust social welfare systems. Findings from this study offer intriguing opportunities for future research, as well as implications for both family-level prevention and interventions. 

### 8.1. Economic Inequities

Empirical research has clearly documented how the adversities for children with a parent in prison stem from intensified economic hardship and consequent family difficulties [26,27,28,29,30,31,32,33]. Consistent with the FIF, findings from the present study support the idea that existing stressors such as material hardship predict family level outcomes. For justice-involved families, material hardship is often a pre-existing condition that both predicts and is worsened by PI [35]. A direct effect was detected between material hardship and youth academic adjustment in preliminary regression analyses (supporting our first hypothesis [H1]), yet the effect was not significant in the SEM analysis. This suggests that psychological well-being of the caregivers mitigates the effects that material hardship otherwise has on youth academic adjustment, lending support for our second hypothesis (H2). The significance of material hardship within this population is consistent with the social causation perspective, in that health and well-being outcomes are dependent on social and economic conditions [44]. Consequently, material hardship may chip away at family resources and resilience, ultimately resulting in poor outcomes. Findings from this study demonstrate that material hardship does negatively impact family resilience processes (i.e., caregiver mental health and social support) as well as youth academic adjustment (H1), yet greater psychological well-being of caregivers may mediate negative outcomes by fostering resilience in these families (H2). The combination of (1) significant indirect effects (i.e., caregiver mental health as a mediating resilience process) and (2) insignificant direct effects between material hardship and youth academic adjustment suggest what Zhao and colleagues (2010) refer to as “indirect-only” mediation [76]. Indirect-only mediation indicates two crucial aspects that lend strength and credibility to our study: (1) the mediator (i.e., caregiver mental health) is consistent with the hypothesized theoretical framework, and (2) it is unlikely that there is an omitted mediator within the model [76]. Taken together, our findings from this study support the FIF in that material hardship appears to be the main conduit to worsening outcomes and that family processes such as sound caregiver mental health likely promote family-level resilience by interrupting the negative pathway of material hardship.

### 8.2. Pathways of Resilience

#### 8.2.1. Positive Parenting

Results indicate that the quality of caregiver parenting skills did not mediate the relationship between material hardship and youth academic adjustment. Interestingly, despite a lack of mediation, there was a significant direct effect in that higher levels of positive parenting skills significantly predicted better overall academic adjustment. Previous research has indicated that, in the context of PI, material hardship is related to more harmful forms of parenting that rely on authoritarian practices [20]. Consistent with Conger’s (2010) work on economic hardship impacting children through parents, this finding may be due to higher levels of economic and psychological stress when one parent is suddenly incarcerated [44] and the parent-at-home adjusts to increased responsibilities with less support [20]. However, a lack of significance between material hardship and positive parenting may indeed still be a sign of resilience. Although it is logical that stress associated with material hardship and cumulative disadvantage might inform the quality of one’s parenting [77], a lack of causal significance may indicate that some parents are able to maintain positive parenting skills despite added contextual stressors. For example, using a within-group analysis, Turney and Wildeman (2013) found that paternal incarceration was associated with increased engagement (i.e., positive parenting) between mothers and their children [47]. It may be too simplistic to categorize parenting practices as “good” or “bad” during PI. In reality, parenting practices are likely far more complex and nuanced. For example, Arditti et al. (2010) found that while more than half of justice-involved mothers in a qualitative study displayed harsh parenting practices, many also demonstrated care and advocacy practices [77]. Together, research on parenting among families experiencing PI suggests that positive parenting scenarios may be possible in conjunction with material hardship experiences that often plague justice-involved families. Findings indicate that parenting practices may indeed remain unchanged, or may even improve, in spite of material hardship conditions. Although this study does not confirm this link, we must conduct further family resilience research with lower socioeconomic status families to continue identifying strengths, with a particular focus on the cultural context of parenting practices.

#### 8.2.2. Social Support

Social support did not appear to yield any mediation effects on the relationship between material hardship and academic adjustment. Nonetheless, analyses did yield noteworthy direct effects. A significant negative relationship was found between material hardship and social support. Indeed, it appears that higher degrees of material hardship significantly predict lower levels of social support. Stack’s (1974) work on kinship networks and social support is helpful in situating the findings from our study [78]. In particular, Stack (1974) emphasizes that networks of social support are not meant to escape poverty per se, as this is considered near impossible on welfare benefits alone, but are meant to help one another survive in the face of material hardship [78]. Furthermore, social support is conceptualized as available only in a quid pro quo state—to the degree that the receiving party also has something to give either immediately or later on [78]. In this way, material hardship may reduce a caregivers’ ability to “give” support, thereby isolating caregivers and limiting the degree to which they are able to receive social support. Meanwhile, an incarcerated parent may incur further loss in material resources (i.e., income) and social commodities. Indeed, findings by Besemer and Dennison (2017) indicate that caregivers of children with an incarcerated parent experienced more social exclusion than the general population and that financial hardship may mediate this relationship [79]. It may be that material hardship prevents social participation broadly, resulting in overall reduced social support. Considering the Danish sociopolitical context may help situate this finding as well. In particular, the Danish welfare and healthcare system is far more generous, accessible, and widespread than that in other Westernized countries [6,7,8,9], potentially rendering social support as a less critical component of safety nets than other parts of the world where welfare resources are scarce. Taken together, previous research and theory may help frame why, in the present study, material hardship was found to predict lower levels of social support. Furthermore, the results from this study highlight how a crisis (i.e., PI) in low-resource (i.e., material hardship) families may interact to result in even lower social support. 

The negative association between material hardship and social support found in this study inspires the question: How do families manage to persevere despite diminished social support? It is noteworthy that despite the significant negative effect material hardship had on social support, there was no significant effect of caregivers’ social support on youth academic adjustment. The lack of association between social support and youth academic adjustment warrants further exploration and may be an indicator of resilience if caregivers are able to prevent social support status from impacting adjustment in school. Although not statistically explanatory of academic adjustment, the relationship between material hardship and social support is concerning, given the stigma and challenges typically associated with justice involvement (i.e., social exclusion), and warrants further empirical exploration to better understand how these constructs contribute to child and family inequality.

#### 8.2.3. Caregiver Mental Health

Perhaps the most noteworthy finding in the present study is both the direct effects, as well as the mediating role, of caregiver mental health symptoms in the relationship between material hardship and youth academic adjustment. It is important to note that caregiver mental health (i.e., symptomology) was not measured using a methodological “gold standard” assessment (e.g., Beck Depression Inventory) but instead relied on self-report of general symptoms (e.g., anxiety) that are indicative of mental health problems. Regardless, results indicate that caregiver mental health was measured well by corresponding indicators. Material hardship significantly predicted caregiver mental health symptoms, indicating that higher levels of material hardship yielded a higher prevalence of mental health symptomology for caregiver respondents. The significant relationship between material hardship and caregiver mental health found in our study is consistent with Conger’s (2010) work in which material hardship is seen as a predictor of stress and poor health outcomes [44]. Furthermore, caregiver mental health symptomology had a significant negative relationship with youth academic adjustment, indicating that higher levels of caregiver-respondent mental health symptoms significantly predicted worse youth academic adjustment. Caregiver mental health symptomology also strongly mediated the effects of material hardship on youth academic adjustment. Considering that the direct effect of material hardship on youth academic adjustment was not significant within the overall model, it is a critical finding that this relationship became significant as a result of the mediating influence of caregiver mental health symptomology. Caregiver mental health as a significant mediating resilience process suggests that material hardship negatively impacts youth academic adjustment through caregiver mental health symptomology. Conversely, caregiver psychological well-being may promote positive youth academic outcomes and may mitigate the effects of material hardship. The mediating influence of caregiver mental health in the present study is consistent with the FIF, in which material hardship is a key stress mechanism that yields a more significant effect on youth academic adjustment when resources and resilience processes are lacking. Furthermore, identifying caregiver mental health as a mediating process highlights an important area of intervention—namely, identifying and addressing both economic stressors, in conjunction with caregiver mental health symptoms, may indeed promote family-level resilience and mitigate academic maladjustment for youth experiencing paternal incarceration. 

### 8.3. Implications

Academic adjustment is an understudied area of focus among youth with an incarcerated parent. Interestingly, our findings indicate that two caregiver factors are significantly associated with youth academic adjustment: (1) positive parenting and (2) sound caregiver mental health. It may be that the common denominator across positive parenting skills and caregiver mental health is the ability to be present and positively engaged with a child’s academic adjustment and progress. Indeed, previous research has demonstrated that the stress of PI on the remaining, non-incarcerated parent can lead to higher stress, increased use of harsh parenting, and role modeling of more antisocial than prosocial behaviors [79,80,81]. Consistent with our findings, positive parenting may be a critical mechanism of promoting positive youth academic adjustment scenarios. Future research should more closely examine the link between the caregiver-at-home, in terms of parenting skills and mental health, and youth academic adjustment. To better understand the nuances of youth academic adjustment, we recommend researchers employ robust measures, such as the Student Engagement in School Four-Dimensional Scale (SES-4DS) [82]. Based on our findings and previous research, we strongly recommend that practitioners, policymakers, and educators consider the primary caregiver of youth with an incarcerated parent. In particular, it may be useful to provide resources (e.g., family therapy, parenting programs, etc.) that promote positive parenting and bolster the mental well-being of the remaining, non-incarcerated caregiver. Furthermore, because youth experiencing PI can feel isolated and require higher levels of support, we recommend that interventions be systemic in nature to address not only the needs of the youth but also the needs of the non-incarcerated caregiver (e.g., mental health and parenting) as an indirect conduit for promoting positive youth academic adjustment and overall family well-being. We further implore researchers and educators to consider specific academic engagement strategies, such as cooperative learning methods [83], that may promote better academic adjustment scenarios and mitigate the school-based vulnerabilities inherent in experiencing PI. 

The most noteworthy implication inherent in our findings is related to the mental health of the non-incarcerated, primary caregiver at home. An abundance of research has demonstrated that the caregivers remaining at home, most often biological mothers in the context of paternal incarceration, experience elevated mental health symptoms [84] and overall increased stress. Indeed, prior research has identified a direct association between caregiver anxiety, depression, and poor youth outcomes during PI [84,85]. Our study adds to this literature and, to the best of our knowledge, is the first to demonstrate that stable caregiver mental health strongly mediates how youth with an imprisoned father fare in school. Furthermore, our findings are in line with other PI scholars who have identified an interactional association between parenting, caregiver mental health, and youth outcomes. We recommend that researchers, policymakers, and practitioners provide direct support (i.e., resources, therapy, etc.) to primary caregivers of youth experiencing PI, based on our findings that, within the context of paternal incarceration, material hardship appears to (1) directly diminish the mental wellbeing (e.g., anxiety, depression, isolation, etc.) and parenting abilities (e.g., fatigue, resorting to harsh parenting, etc.) of caregivers at home and (2) indirectly impact youth academic adjustment through the primary caregiver. In this way, we implore PI scholars and practitioners not only to look at the youth impacted but also to consider the many systems (e.g., school, home, family, etc.) that indirectly shape the experiences and well-being of youth with an imprisoned father. Attending to the well-being of the youth alone will likely only help so much if the primary caregiver is not supported as well. Indeed, supporting the primary caregiver at home may be one of the most important and encompassing interventions for mitigating the effects of PI on families. Nevertheless, specific interventions for caregivers of youth with an incarcerated parent have yet to be extensively researched and implemented. We join other PI scholars [84] in recommending that future research and practice focus on identifying, developing, and testing intervention strategies that support primary caregivers of youth impacted by PI, and particularly focus on mental health and parenting to promote positive family adjustment scenarios. 

### 8.4. Limitations and Future Research

The primary limitation in the present study was its reliance on secondary data analysis. While the analytic model was informed by extant theory and literature, the model was somewhat constrained by the items available in the original survey data source. Because this study aimed to provide a preliminary empirical testing of the FIF, it would have been ideal to include variables such as family instability and protective factors, as well as more comprehensive parenting, mental health, and youth outcome measures. Similarly, many survey items were dichotomous (i.e., “yes”/”no” answers), constraining the degree of variability in the analysis. Future research would benefit from increased variability (i.e., Likert-scale responses) as well as qualitative data to better capture the nuances of these variables. Additionally, we were unable to consider certain demographic information, such as income level, employment status of the caregiver respondent, paternal carceral sentence length, type of incarceration, etc., that might have provided important group-level information. Furthermore, the survey relied on self-report as the primary method of data collection. Although this gave voice to respondents, relying solely on self-report indicators may compromise internal validity. Future research should expand on the present study by incorporating data triangulation methods such as “gold standard” assessments (e.g., Beck Depression Inventory) as well as in-depth qualitative interviews to produce thick descriptions of these phenomena. 

The original survey was constructed in Danish. Prior to data analysis, the survey was translated from Danish to English by a bilingual researcher employed by the primary data source (i.e., VIVE). It is possible that slight dialectical translation errors occurred and/or were not identified and resolved. While we attempted to resolve these translation errors to the best of our ability, it is possible that discrepancies were missed as a result of dialect nuances. Despite these limitations, data of these kind rarely exist, and the results from this study are an important first step in the next wave of PI research using a resilience framework for examining family processes. Multiple directions for future research exist. First, although the indirect effect was approaching significance, it was surprising to find that social support was not identified as a mediating resilience process. Future research should examine this relationship more closely, perhaps identifying better ways to measure social support as a construct and examining social support within sociopolitical contexts (e.g., social welfare policies). Additionally, caregiver mental health was identified as a critical resilience process. Future research should attempt to expand on the findings from this study—identifying precise ways in which caregiver mental health can promote risk and/or resilience in families impacted by paternal incarceration. Similarly, we recommend future research examine whether this mediating relationship holds across other data sets, particularly in countries where material hardship may be more severe and mental health support, among other social safety net resources, may be lacking. Furthermore, it is possible that a few non-significant direct effects are representative of resilience, highlighting the potential for caregivers to prevent adverse experiences from impacting youth academic adjustment. It is unclear the degree to which these relationships are, or are not, indicative of resilience processes. 

The results that were found in this study are inextricable from the sociopolitical context in Denmark. Nonetheless, we contend that significant findings from a country with robust socialized resources are likely to hold true in countries with worse conditions and less socialized support, such as the U.S., and may well inform future research in other developed countries [6,7,8,9]. Future research should examine these effects more closely to identify how components of the FIF manifest under paternal incarceration conditions. While scholars have recently called for increased use of within-group analyses when examining family resilience processes in PI [31,33], future research may also benefit from a between-groups moderated-mediation analysis that examines how resilience processes compare with families unaffected by PI. Finally, although this research highlights risk and resilience processes for families while a parent is incarcerated, a critical area for future research is examining how risk and resilience processes change as incarcerated parents find their way home, and the process by which formerly incarcerated parents navigate community reentry and family reunification.

## 9. Conclusions

Although carceral confinement is best understood in the context of a country’s sociocultural factors, criminal justice policies, and carceral practices, research indicates that PI yields an abundance of risks for families globally. Our study adds to a burgeoning body of international research examining potential resilience processes of families impacted by incarceration. Of note, our findings suggest that stable caregiver mental health plays an important role for overall family wellness by mitigating the effects of material hardship and promoting positive youth academic scenarios. Our findings robustly advance our understanding of what may help families rise above the challenges of PI. As incarceration practices continue to plague families around the world, we implore the many interdisciplinary scholars examining the broad effects of PI to continue identifying and honoring the strengths and resilience factors that families with an imprisoned parent may leverage to fare well when a loved one is incarcerated.

## Figures and Tables

**Table 1:** Description of Data.

		*N*	Mean	St. Dev	Skewness	Kurtosis	Min.	Max.
**Material Hardship**								
	Financial Problems	685	1.50	2.143	1.253	0.292	0	7
	Basic Needs	690	0.63	1.044	1.485	0.927	0	4
	Overall Finances	682	2.62	0.883	0.006	−0.480	1	5
**Positive Parenting**								
	Notice Behavior	719	4.44	0.591	−0.741	1.154	1	5
	Praise Task	719	4.56	0.587	−1.190	1.956	1	5
	Praise Behavior	719	4.43	0.694	−1.005	0.639	1	5
**Caregiver Mental Health**								
	Anxiety	703	0.26	0.437	1.118	−0.750	0	1
	Insomnia	702	0.46	0.499	0.154	−1.976	0	1
	Overwhelmed	701	0.30	0.458	0.875	−1.234	0	1
**Social Support**								
	Parents	696	3.26	1.618	−0.218	−1.563	1	5
	Siblings	696	3.15	1.396	0.003	−1.315	1	5
	Relatives	696	2.91	1.365	0.195	−1.209	1	5
	Friends	696	3.59	1.209	−0.411	−0.885	1	5
	Neighbors	696	2.69	1.185	0.562	−0.611	1	5
**Youth Academic Adjustment**								
	Adjustment	689	8.40	1.829	−1.413	1.667	2	10
	Concentration	712	2.19	0.701	−0.273	−0.956	1	3
	Social Inclusion	712	5.30	1.114	−1.531	1.390	2	6

**Table 2:** Variable Correlation Matrix.

Variable	1	2	3	4	5
Material Hardship	1.00				
Positive Parenting	0.637 **	1.00			
Caregiver Mental Health	0.644 **	0.564 **	1.00		
Social Support	0.566 **	0.500 **	0.902 **	1.00	
Academic Adjustment	0.491 **	0.44 **	0.794 **	0.880 **	1.00

** Correlation is significant at the 0.01 level (2-tailed).

**Table 3:** Parameter Estimates for the Final Model.

Variable	Maximum Likelihood Estimates	*t-*Score Value *	SE	Standardized Estimates †	R^2^ ‡
**Material Hardship**					
Financial Problems	1.000	--	--	0.830	0.689
Basic Needs	0.348	13.136 ***	0.027	0.593	0.351
Overall Finances	0.318	11.321 ***	0.028	0.640	0.409
**Positive Parenting**					
Notice Behavior	1.00	--	--	0.638	0.407
Praise Task	1.374	13.997 ***	0.098	0.883	0.779
Praise Behavior	1.481	13.706 ***	0.108	0.805	0.647
**Caregiver Mental Health**					
Anxiety	1.000	--	--	0.607	0.368
Insomnia	1.088	9.862 ***	0.110	0.578	0.334
Overwhelmed	1.216	9.569 ***	0.127	0.703	0.494
**Social Support**					
Parents	1.000	--	--	0.451	0.204
Siblings	1.036	10.611 ***	0.098	0.542	0.293
Relatives	1.090	7.677 ***	0.142	0.583	0.340
Friends	1.078	5.394 ***	0.200	0.651	0.424
Neighbors	0.874	4.879 ***	0.179	0.539	0.290
**Youth Academic Adjustment**					
Adjustment	1.000	--	--	0.748	0.559
Concentration	0.349	8.913 ***	0.039	0.680	0.463
Social Inclusion	0.415	9.097 ***	0.046	0.510	0.260

† Standardized estimates closer to 0.9 are better. ‡ R2 estimates above 0.3 are preferred. * Correlation is significant at the 0.05 level (2-tailed). *** Correlation is significant at the 0.001 level (2-tailed).

**Table 4:** Structural Model Standardized Coefficients.

Variable	*R*^2^ (Structural)	Direct Coefficients	Direct *t*-Score *	Indirect Coefficients	Indirect *t*-Score
**Material Hardship**					
Positive Parenting		0.003	−0.195	0.000	0.055
Caregiver Mental Health		0.544	10.622 ***	−0.164	−3.082 **
Social Support		−0.178	−3.365 **	−0.009	−0.808
Youth Academic Adjustment		−0.014	−0.175		
**Positive Parenting**	0.000				
Material Hardship					
Caregiver Mental Health					
Social Support					
Youth Academic Adjust.		0.115	2.277 *		
**Caregiver Mental Health**	0.296				
Material Hardship					
Positive Parenting					
Social Support					
Youth Academic Adjustment		−0.301	−3.458 **		
**Social Support**	0.032				
Material Hardship					
Positive Parenting					
Caregiver Mental Health					
Youth Academic Adjustment		0.051	0.852		
**Youth Academic Adjustment**	0.123				
Positive Parenting					
Caregiver Mental Health					
Social Support					

* Correlation is significant at the 0.05 level (2-tailed). ** Correlation is significant at the 0.01 level (2-tailed). *** Correlation is significant at the 0.001 level (2-tailed).

## Data Availability

Restrictions apply to the availability of these data. Data were obtained from VIVE, the Danish Center for Social Science Research and are only available at vive.dk/en/welcome/ with the permission of VIVE and the Danish Ministry of the Interior and Housing.
